# Estimating carbon leakage from aviation by combining sectoral and general equilibrium models

**DOI:** 10.1016/j.mex.2024.102975

**Published:** 2024-10-03

**Authors:** Taoyuan Wei, Steffen Kallbekken

**Affiliations:** CICERO Center for International Climate Research, P.O. Box 1129, Blindern, NO-0318 Oslo, Norway

**Keywords:** Climate mitigation, Sector model, Aviation model, CGE model, Sustainable aviation fuels, Energy taxation directive, Emissions trading scheme, Scenario analysis, Transportation, Carbon emissions, European Green Deal, A method of estimating carbon leakage from aviation based on combining sectoral and general equilibrium models

## Abstract

This article describes a procedure for estimating carbon leakage from policies targeting aviation based on alternative scenarios. The key innovation to ensure greater robustness is that all scenarios are simulated by two different types of models: a sectoral model for aviation and a computable general equilibrium model.

• The implementation of scenario simulation in both models is explained
• The calculation of carbon leakage is explained step by step

## Graphical abstract

**Figure:**
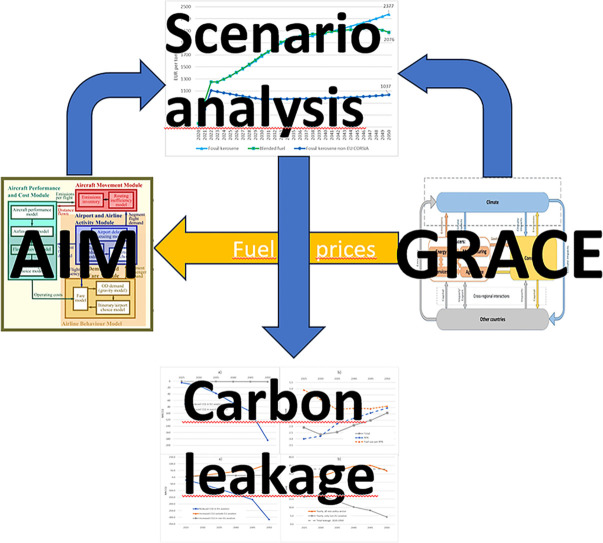

Specifications table

**Table:** 

Subject area:	Economics, Econometrics and Finance; Energy; Environmental Sciences; Social Sciences
More specific subject area:	Applied economics; Economic modeling; Transportation economics; Regional economics; Computable general equilibrium (CGE) modeling; Energy modeling; Scenario analysis; Climate mitigation
Name of your method:	A method of estimating carbon leakage from aviation based on combining sectoral and general equilibrium models
Name and reference of original method:	1. The sectoral model for aviation (AIM). Dray L. Aviation Integrated Model Open Source [Code]. 2022 [cited 2022 7 July]Available from: http://www.atslab.org/aviation-integrated-model-open-source-code/ 2. The CGE model GRACE. Aaheim A, Orlov A, Wei T, Glomsrød S. GRACE model and applications. Report. Oslo, Norway: CICERO; 2018. Report No.: 05.
Resource availability:	Data and code associated with this article are available at https://doi.org/10.17632/knpsd733ww.1. The GTAP_power database version 10 is available at https://www.gtap.agecon.purdue.edu.

## Background

The method presented in this article was used to study carbon leakage from aviation under the European Union fit for 55 (FF55) policies [16], but the method is flexible and could be applied to a broader set of policies targeting emissions from aviation in different regions. This article provides supplementary details about how the method is implemented in the sectoral and general equilibrium models used by Wei and Kallbekken [16]. The method can highlight the potential differences between the models by scenario analysis. This article starts with two well-developed models and describes how carbon leakage in alternative scenarios is calculated step by step to illustrate how sectoral and general equilibrium models can be used together to arrive at economic and policy insights.

## Method details

To facilitate the understanding of the method, the explanatory part of the method details is largely taken from Wei and Kallbekken [16]. Carbon leakage is defined as “the increase in CO_2_ emissions outside the economic sectors targeted by a mitigation action divided by the reduced emissions within the targeted sectors” [16], i.e.,

(1) $$Leakage=\frac{-\Delta CO_{2,\;outside\;policy\;sector}}{\Delta CO_{2,\;within\;policy\;sector}}\times \;100\%$$

The uniqueness of the method is that two global models are used to numerically estimate the carbon leakage in the aviation sector: A sectoral model is used to calculate carbon leakage within the aviation market and a computable general equilibrium (CGE) model covering all sectors is used to calculate carbon leakage by considering CO_2_ emissions across all sectors outside the policy sector, including non-aviation sectors.

### The sectoral model AIM

**Fig. 1:**
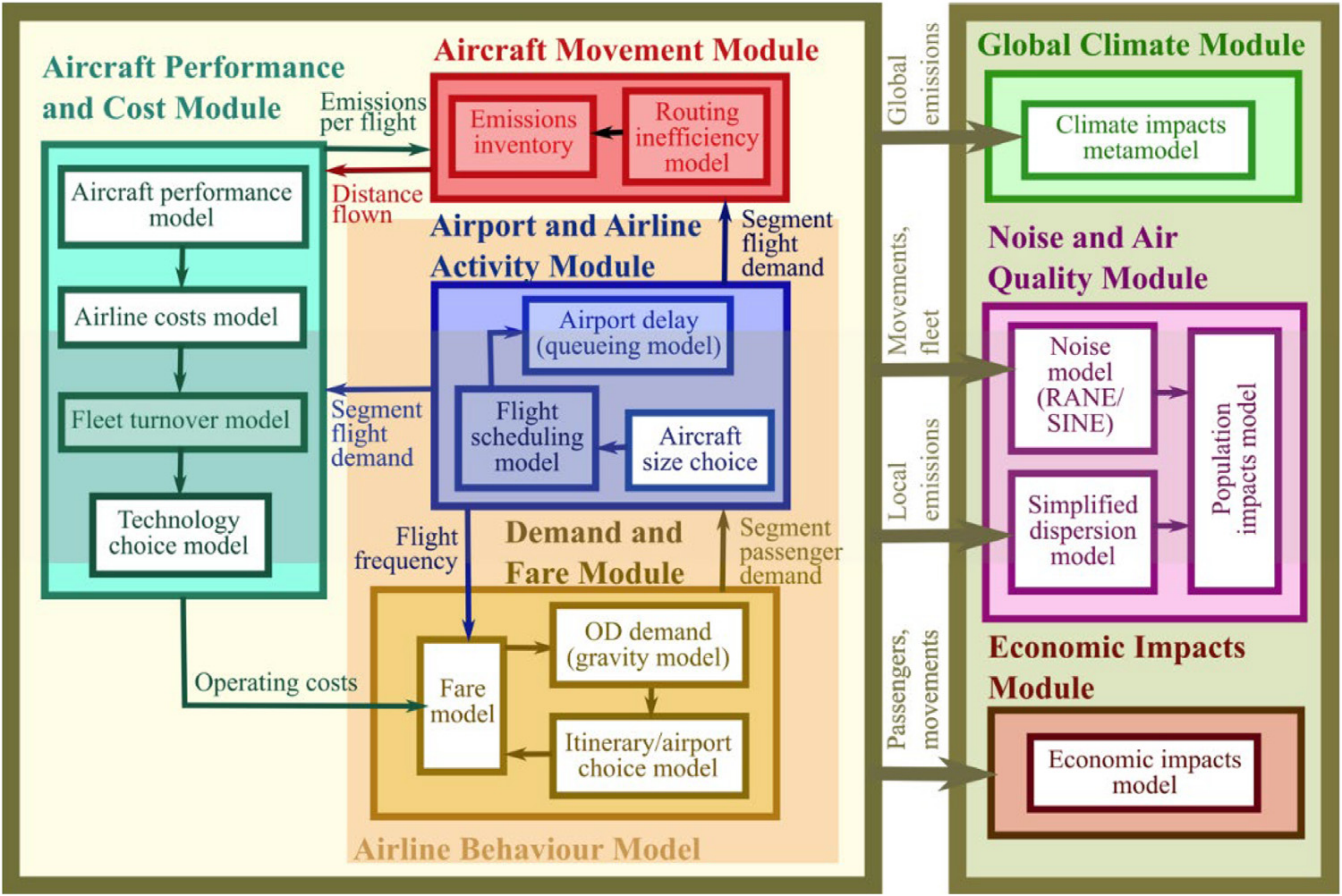
Detailed sub-modules of AIM 2015. Source: Dray [3].

The open-source global aviation systems model AIM [5] simulates “interactions between passenger demand, airline costs and behavior, airport capacity, fleets, technology availability and policy” [4]. Fig. 1 below shows in detail the structure of the AIM 2015 version. For our purpose of estimating carbon leakage, we run only the modules on the left panel of Fig. 1. Full documentation is available online [3].

We use the AIM model to assess how the FF55 policies affect global aviation emissions and economic factors from 2015 to 2050. To do so we first run the model assuming regional population to grow in line with the medium projection from “World Population Prospects 2022” [15] and the regional economies to grow in line with the GDP forecast by OECD [12]. We then estimate how FF55 policies would alter costs, changing in turn airline and passenger behaviour in the model.

### The CGE model GRACE

**Fig. 2:**
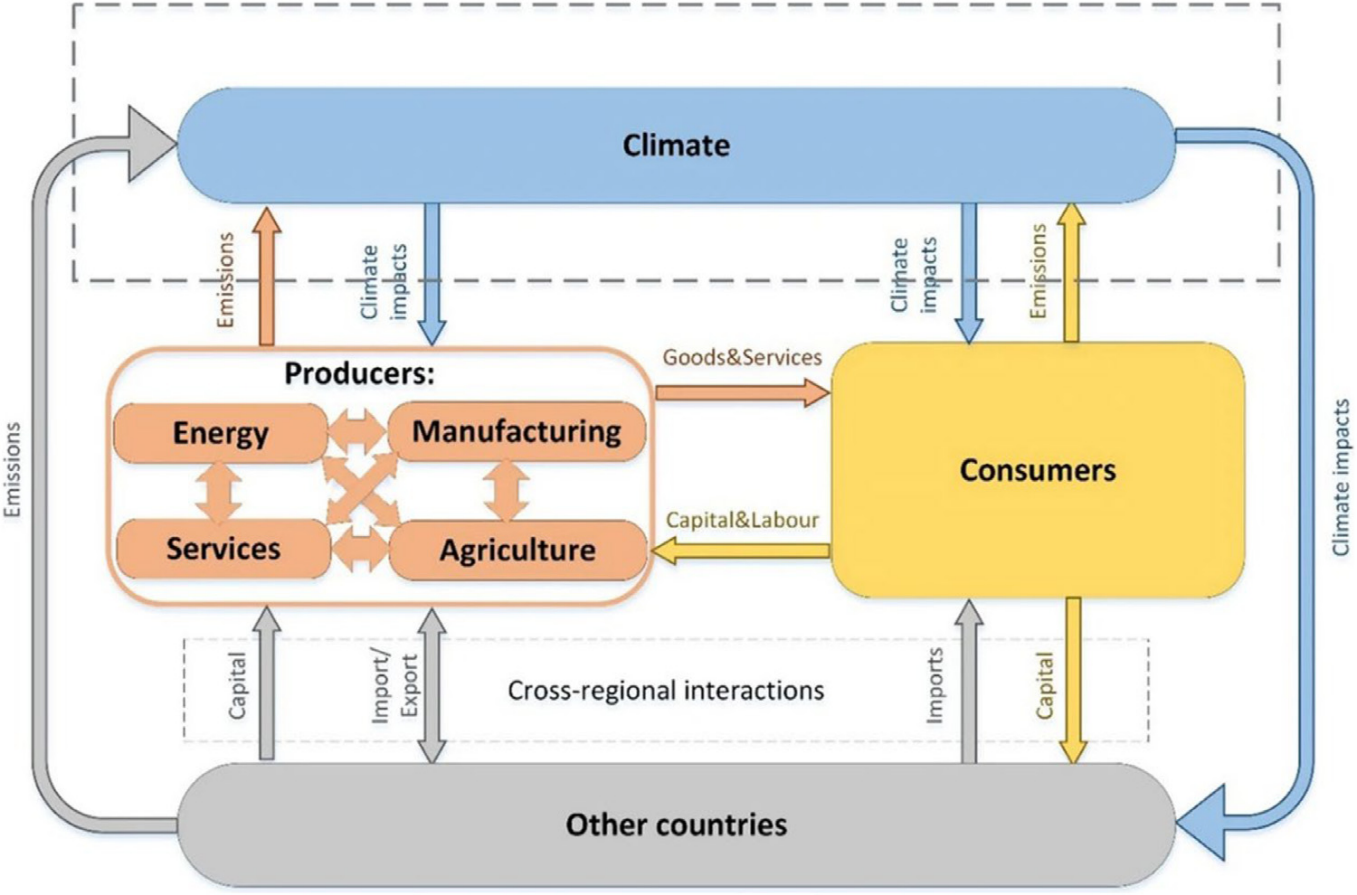
Circular flows of economic activities in GRACE. Source: Orlov et al. [13].

GRACE is a multi-sector, multi-regional, recursively dynamic CGE model [1]. The version of the model used in this study has four regions: The EU region (EEA members, the UK, and Switzerland); CORSIA Phase 1 region (CORSIA countries participating in Phase 1), CORSIA Phase 2 region (only CORSIA countries participating in Phase 2), and the rest of the world. Within each region, production takes place in ten sectors: Five sectors to produce energy (coal, gas, crude oil, refined oil, and power), two sectors to provide transportation services (aviation and all the other transport), and three other aggregated sectors (agriculture, manufacturing, and other services). Fig. 2 shows the flows of economic activities in the model. Further details about GRACE are provided in Aaheim et al. [1].

Fossil kerosene production cannot be disaggregated as a separate sector in GRACE. To overcome this, we approximate fossil kerosene by “refined oil”. The policies are implemented in GRACE as follows. The ReFuelEU SAF requirement is met by introducing a new sector to produce the SAF needed by aviation in the EU at a given price, to replace refined oil. This means that for a given level of SAF used in the sector, an equivalent reduced amount of refined oil is used. Carbon pricing (fuel taxes required by ETD plus the EU ETS) is treated as taxes on relevant fuels used by the aviation sector in the EU region.

### Data sources

This study relies on data gathered from several sources. The sources of the base year data used by the sectoral model AIM were described in the online model document [3]. The base year data used by the CGE model GRACE was taken from the GTAP (Global Trade Analysis Project) database version 10 [2]. Projection of population growth was taken from UNPD [15]. Projection of Income growth was indicated by GDP per capita, where data of long-term real GDP were from OECD [12]. The price changes of fossil kerosene were calculated from the crude oil prices in history [10] and in the future as used in the ‘Stated Policy Scenario (STEPS)’ of IEA [9].

The mix of SAF and fossil kerosene that goes into the blended fuel used by the EU aviation sector is taken from ReFuelEU Aviation [8]. Historical EU ETS prices are from World Bank [17], whereas estimated future EU ETS prices are from the Impact Assessment Report of the EU Climate Target Plan [6] in one scenario and are based on SEO & NLR [14] in another scenario. Future energy taxes are based on the proposed revision of ETD policy [7], and, finally, future SAF prices are from SEO & NLR [14].

### Scenarios

There are two main scenarios in our study. The first is a “NoPolicy” scenario where no new policies are introduced. This serves as a baseline to quantify the carbon leakage induced by the new policies. In the second scenario, called “Policy”, policies matching as closely as possible the specific FF55 policies targeting aviation, are introduced in both models. As a robustness check we run additional simulations with both models where we change key assumptions.

### Implementation

All necessary code and data for this article can be found at Mendeley^1^, where a ZIP file contains three folders: ‘AIMModel_Free,’ ‘Data,’ and ‘GRACE.’ The folder ‘AIMModel_Free’ includes all files of the sectoral model of aviation AIM version 9 downloaded from Dray [3]; The folder ‘GRACE’ includes all files of the GRACE model used for Wei and Kallbekken [16]; and the folder ‘Data’ includes the files of data related to both models to calculate carbon leakage in aviation under the EU fit for 55 policies. Below we describe in turn how to run both models.

### Run the AIM model

We replaced only two files in the public version of the AIM model with our own: ‘AIMRunParameters.csv’ in the subfolder ‘\v9’ and ‘ScenarioData.csv’ in the subfolder ‘\v9\data_2015baseyear_free.’ In the new file ‘AIMRunParameters.csv,’ we define parameters for nine scenarios that were used in Wei and Kallbekken [16]. The correspondence between them is listed in Table 1.

**Table 1:** The correspondence between scenarios in the code file ‘AIMRunParameters.csv’’ and Wei and Kallbekken [16].

RunNumber and RunID in code	Name in Wei and Kallbekken [16]
2 TestBaseDataRFree_SSP2_C	Main scenario ‘NoPolicy’
3 TestBaseDataRFree_SSP2_C0	Modified ‘NoPolicy’ where no carbon tax is implemented in all CORSIA countries.
4 TestBaseDataRFree_SSP2_C5	Modified ‘NoPolicy’ where the carbon tax implemented in all CORSIA countries is five times the main ‘NoPolicy’ level.
5 TestBaseDataRFree_SSP2FF55AB	Main scenario ‘Policy’
6 TestBaseDataRFree_SSP2FF55AB0	Modified ‘Policy’ where no carbon tax is implemented in all CORSIA countries.
7 TestBaseDataRFree_SSP2FF55AB5	Modified ‘Policy’ where the carbon tax implemented in all CORSIA countries is five times the main ‘NoPolicy’ level.
8 TestBaseDataRFree_SSP2FF55B	Scenario ‘Only Biofuel B’ where only biofuel B is used in the blended oil in aviation.
9 TestBaseDataRFree_SSP2FF55A	Scenario ‘Only Biofuel A’ where only biofuel A is used in the blended oil in aviation.
10 TestBaseDataRFree_SSP2FF55ABP	Scenario ‘Lower fossil kerosene price’ with the global fossil kerosene price adjusted by following the price path in the ‘Rest of World’ in the main ‘Policy’ scenario generated from the CGE model GRACE

The parameters of these scenarios are updated in the file ‘ScenarioData.csv, ’ a copy of an Excel file with the same file name (‘scenarioData.xlsx’) in the same folder. The input data to the Excel file are mainly presented in the file ‘SimulationScenarios.xlsx’ (in the folder ‘Data’), where Figures 3-7 in Wei and Kallbekken [16] are generated. The input data of population and GDP growth are taken from two files ‘WPP2022.xlsx’ and ‘GDP_OECD.xlsx’ in the folder ‘GRACE.’ The input data sources and processing have been explained in Wei and Kallbekken [16].

After replacing the two files, we run the AIM model by executing the file ‘AIMModel_v9.java’ in the JAVA SE JDK 17.0.11 environment. The simulation results are shown in the folder ‘\v9\output’ with the name of each scenario as a subfolder. By default, there are several files of results for each scenario. To calculate further the carbon leakage from aviation, we use the results from the file of e.g. the main ‘Policy’ scenario, ‘SummaryData_TestBaseDataRFree_SSP2FF55AB.csv,’ which is copied to a sheet ‘FF55AB’ in an excel file ‘CLeakageBioABMain.xlsx’ in the folder ‘Data.’ Similarly, the file of the main ‘NoPolicy’ scenario, ‘SummaryData_TestBaseDataRFree_SSP2_C.csv,’ is copied to a sheet ‘SSP2’ in the same Excel file ‘CLeakageBioABMain.xlsx.’ Then, the carbon leakage is automatically calculated in the sheet ‘CO2LeakageRedifine,’ where Figure 9 in Wei and Kallbekken [16] is shown. In the same Excel file, Figure 12 in Wei and Kallbekken [16] is shown in the sheet ‘DecompositionRedine.’ To calculate (or update) the carbon leakages in the alternative scenarios, we copy the results in the file starting with ‘SummaryData’ for those scenarios to the corresponding Excel files ‘CLeakage0CORSIACTax.xlsx,’ ‘CLeakage5TimesCORSIACTax.xlsx,’ ‘CLeakageOnlyBioA.xlsx, ’ ‘CLeakageOnlyBioB.xlsx, ’ and ‘CLeakageBioABMainLowPris.xlsx. ’

### Run the GRACE model

All files of the GRACE model [1] used by this article are located in the folder ‘GRACE.’

First of all, we aggregate the 2014 data in the Global Trade Analysis Project (GTAP) database v10 [2] into one file ‘aviate.gdx,’ where the world is divided into four regions: The EU region (EU); CORSIA Phase 1 region (OCS1); CORSIA Phase 2 region (OCS2); and the rest of the world (ROW). For each region, the economy includes ten production activities including five energy sectors (coal, crude oil, gas, refined oil, and electricity), two transportation sectors (aviation and all the other transport), agriculture, manufacturing, and services. The ‘aviate.gdx’ is then called by the file ‘GTAP_input_main.gms.’

Two stages of calibration and simulation are needed to prepare the main ‘NoPolicy’ scenario in GRACE. To calibrate the main ‘NoPolicy’ scenario, we need the same population and GDP growth data as used in the AIM model. Hence, we copy the ‘ScenarioData.xlsx’ in the AIM model folder to the folder ‘GRACE’ and transform it to the GDX format as ‘ScenarioData.gdx,’ where the data of population and GDP growth are extracted and then saved as ‘AIMSSP.gdx’ by running the code file ‘AIM2015SSP.gms.’ The ‘AIMSSP.gdx’ file is called by the file ‘scenarios_declare.gms’ to introduce the population and GDP growth data into the GRACE model. In the calibration stage, the population and GDP growth data are set exogenous for the main ‘NoPolicy’ scenario by making total factor productivities (TFP) as endogenous. In the simulation stage, the calibrated TFP values together with other exogenous parameters are saved in a file ‘efftfp.gdx,’ which is called as exogenous parameters to reproduce the ‘NoPolicy’ scenario, where the GDP becomes endogenous. The same file ‘efftfp.gdx’ is called to introduce the same exogenous parameters for all the other scenarios, including the main ‘Policy’ scenarios.

We run the same file ‘core.gms’ for both the calibration and simulation stages. In the calibration stage, we make two parameters to be true, i.e., ‘calib_toggle = 1; calib_GDP(r) = 1;’ in the file ‘core.gms’ and then run the file in the GAMS 44.4.0 environment. In the simulation stage, we make two parameters to be false, i.e., ‘calib_toggle = 0; calib_GDP(r) = 0;’ in the file ‘core.gms’ and then run the file in the GAMS 44.4.0 environment.

All the other scenarios than the main ‘NoPolicy’ scenario have different fuel prices and carbon taxes in the aviation sector. The price of the blended fuel in the EU aviation sector in the ‘Policy’ scenarios is calculated from the fuel shares and taxes introduced by the EU FF55 policies. The price of the blended fuel is represented by the parameter ‘Prisfuel’ in the file ‘’scenarios_declare.gms.’

The results of the main ‘NoPolicy’ and the main ‘Policy’ scenarios shown as the respective scenarios ‘BAU’ and ‘SN1’ in the file ‘aviate_res.gdx’ are obtained by running the ‘core.gms’ file in the GAMS 44.4.0 environment. We rename the resulting file to be ‘aviate_resMain.gdx’ to avoid it being rewritten by repeatedly running the model for the other scenarios. Similarly, we rename the resulting files for the other scenarios obtained below.

To obtain results for other alternative scenarios, we activate different calculations of relevant parameters in the file ‘scenarios_declare.gms.’ To obtain results for the case where no carbon tax is implemented in all CORSIA countries, we activate one line in the file ‘scenarios_declare.gms.’

CPris(r,SIM,t)$(not sameas(r,'EU') and CPris(r,SIM,t) and (ord(t)≥7)) = CPris(r,SIM,' 2020′);

Similarly, To obtain results for the case where the carbon tax implemented in all CORSIA countries is five times the main ‘NoPolicy’ level, we activate one line in the file ‘scenarios_declare.gms.’

CPris(r,SIM,t)$(not sameas(r,'EU') and CPris(r,SIM,t) and (ord(t)≥7)) = CPris(r,SIM, t)*5;

To obtain results for the case where only biofuel B is used in the blended oil in aviation., we deactivate the two lines for the above two alternative scenarios and activate two lines in the file ‘scenarios_declare.gms.’

Prisfuel(t,'BioAB') = Prisfuel(t,'BioB') * Sfuel(t,'BioAB') + Prisfuel(t,'SAF') * Sfuel(t,'SAF')+ Prisfuel(t,'JETA') * Sfuel(t,'JetA');

Pris(r,'SN1′,t)$(ord(t)≥7) = (Prisfuel(t,'BioAB')/Prisfuel(t,'JetA')-Sfuel(t,'JetA'))/ (1-Sfuel(t,'JetA'));

Similarly, to obtain results for the case where only biofuel A is used in the blended oil in aviation., we deactivate the two lines for the above alternative scenarios and activate two lines in the file ‘scenarios_declare.gms.’

Prisfuel(t,'BioAB') = Prisfuel(t,'BioA') * Sfuel(t,'BioAB') + Prisfuel(t,'SAF') * Sfuel(t,'SAF')+ Prisfuel(t,'JETA') * Sfuel(t,'JetA');

Pris(r,'SN1′,t)$(ord(t)≥7) = (Prisfuel(t,'BioAB')/Prisfuel(t,'JetA')-Sfuel(t,'JetA'))/ (1-Sfuel(t,'JetA'));

All the resulting files are then used to calculate carbon leakage in aviation in the file ‘resultsGRACE20231115.xlsx’ located in the folder ‘Data.’ For each scenario, we copy the necessary results from the resulting GDX files starting with ‘aviate_res’, including the CO_2_ emissions from fossil fuels as regional total (‘EMSTOTr_T’) and by fossil fuel in aviation (‘EMSTOTSr_T’), Exports of aviation services from EU (‘EXRr_T’), and total domestic aviation services in the regions other than EU (‘XDr_T’). After these results data are copied, the carbon leakage in aviation is automatically calculated in the file ‘resultsGRACE20231115.xlsx.’ The deviations in refined oil prices in the main ‘Policy’ scenario from the main ‘NoPolicy’ scenario are calculated in the sheet ‘PrisRefinedOil’ after the results data of refined oil prices (‘P_T’) are copied from the results file ‘aviate_resMain.gdx.’

In the file ‘resultsGRACE20231115.xlsx’, we can find Figure 10 of Wei and Kallbekken [16] in the sheet ‘PrisRefinedOil,’ Figure 11 of Wei and Kallbekken [16] in the sheet ‘CleakMain,’ and Figure 13 of Wei and Kallbekken [16] in the sheet ‘Fig13.’

## Method validation

In an earlier version of Wei and Kallbekken [16], the carbon leakage from aviation was calculated based on the population and GDP growth data from the SSP2 scenario [11]. This scenario was created around ten years ago. For both models, the estimated carbon leakage in that version was similar to what appears in the final version of Wei and Kallbekken [16]. This is an indication that the results are relatively robust across a substantial range of parameter values for population growth and GDP growth.

## Limitations

Several limitations have been mentioned in Wei and Kallbekken [16]. A notable challenge is that the sectoral model does not consider substitution between aviation and other transport options, which the CGE model shows to be essential for estimating the full extent of carbon leakage. The simulation of the future market of SAFs can be improved, including to take capacity constraints into account, as well as to represent different production pathways for SAFs with different prices and different emissions. For GRACE a more detailed representation of the aviation sector would be very helpful when conducting joint analyses with a sectoral aviation model.

## Ethics statements

The author declares that this work involved no ethics issues.

## CRediT authorship contribution statement

**Taoyuan Wei:** Methodology, Software, Writing – original draft, Writing – review & editing. **Steffen Kallbekken:** Conceptualization, Writing – review & editing, Funding acquisition.

## Declaration of competing interest

The authors declare that they have no known competing financial interests or personal relationships that could have appeared to influence the work reported in this paper.

## Data Availability

We have shared our data at Mendeley as indicated in the manuscript.

## Footnotes

1 https://doi.org/10.17632/knpsd733ww.1.
